# Hydrophilic polymer coating delamination during neurointerventional treatment after microcatheter withdrawal: particulate identification through attenuated total reflection Fourier-transform infrared spectroscopy

**DOI:** 10.3389/fneur.2024.1479375

**Published:** 2025-01-15

**Authors:** Sebastian J. Müller, Pablo Albiña-Palmarola, Stefan Konieczny, Gregor Manke, Sebastian Fischer, Hans Henkes

**Affiliations:** ^1^Neuroradiologische Klinik, Kopf- und Neurozentrum, Klinikum Stuttgart, Stuttgart, Germany; ^2^Neuroradiologische Klinik, Universitätsklinikum Magdeburg, Magdeburg, Germany; ^3^IFAS “Institut für Qualitätssicherung und angewandte Schadensanalyse” GmbH, Dortmund, Germany; ^4^Euro-Labor GmbH “Institute for Failure Analysis and Materials Investigation”, Bochum, Germany; ^5^Klinik für diagnostische und interventionelle Radiologie, Neuroradiologie und Nuklearmedizin, Knapp-schaftskrankenhaus Bochum Langendreer, Ruhr Universität Bochum, Bochum, Germany; ^6^Klinik für Radiologie und Neuroradiologie, Klinikum Siegen, Siegen, Germany; ^7^Medizinische Fakultät, Universität Duisburg-Essen, Essen, Germany

**Keywords:** polymer coating, hydrophilic coating embolization, endovascular treatment, attenuated total reflection, Fourier-transform infrared spectroscopy, microcatheter

## Abstract

Hydrophilic coating embolism (HCE) is a rare and underreported complication in neurointerventional practice that can lead to serious medical consequences. Two endovascular procedures were interrupted at our institution after a cloudy liquid content was observed inside the rotating hemostatic valves (RHV) during microcatheter withdrawal. In both cases, the same type of microcatheter (Prowler Select Plus) and RHV (Merit) were being used, and coating dislodgement was suspected. Attenuated total reflection Fourier-transform infrared spectroscopy (ATR-FTIR) was used to identify the nature of such debris and compared it to samples obtained from different parts of an unused microcatheter and RHV. In an independent second analysis, an *in vitro* simulation of the withdrawal maneuver was conducted, followed by ATR-FTIR analysis. During both *in vivo* and *in vitro* observations, the presence of polyvinylpyrrolidone, a hydrophilic polymer commonly used for intravascular devices manufacture, was confirmed inside the RHV, and its origin was traced back to the surface coating of the distal and middle portions of the Prowler Select Plus microcatheter. This constitutes the first clinical report where hydrophilic coating dislodgement is linked to the microcatheter withdrawal maneuver using a specific microcatheter type, further replicated in an *in vitro* setting.

## 1 Introduction

Since the mid-1980s, hydrophilic polymer coatings have been increasingly used on a wide range of intravascular medical devices such as guidewires, catheters, sheaths, stents, and coils. Hydrophilic polymers, such as polyvinylpyrrolidone, polyacrylamide, and polyoxyethylene, absorb water and expand when in contact with aqueous environments like saline and blood. This expansion increases the device’s lubricity, resulting in enhanced hemocompatibility and maneuverability, while decreasing the risk of vessel injury, dissection/vasospasm, and thromboembolic complications (1, 2). Their use has revolutionized the fields of interventional neuroradiology, interventional cardiology, and vascular surgery; however, since 1997, cases with embolism secondary to coating material delamination have emerged (3–6), prompting the US Food and Drug Administration to issue a warning concerning this apparently rare complication (7). The full impact of hydrophilic coating embolism (HCE) is still uncertain. This is partly due to obstacles related to reporting and publishing processes, as well as the difficulty in recognizing it through naked eye observation. Furthermore, targeted histopathological or spectroscopic recognition is not frequently performed (8). While the available data is limited, some studies have reported autopsy rates as high as 13% (9), and despite advances in coating technology, new cases continue to be reported every year (5, 10–12).

The factors contributing to coating delamination are likely multifactorial; however, certain aspects related to device manufacture and manipulation have been identified, including some techniques and maneuvers used in routine neurointerventional practice (3, 6, 10, 13–17). Moreover, this phenomenon can lead to several medical complications that may result in neurological sequelae or even death. Therefore, physicians should be aware of this unreported phenomenon and, if suspected, thoroughly evaluate the potential causes involved (18).

This study describes two neurointerventional procedures where a cloudy content, indicating potential coating delamination, was observed inside their respective rotating hemostatic valves (RHVs) after a specific type of microcatheter was withdrawn through the valve’s main port. The primary goal was to analyze the molecular composition of the abnormal debris using attenuated total reflection Fourier-transform infrared (ATR-FTIR) spectroscopy and to determine its source. Additionally, the study aimed to evaluate whether a similar phenomenon occurred in an *in vitro* simulation designed to mimic the microcatheter withdrawal maneuver.

## 2 Materials and methods

### 2.1 Patients

The first case involved a 40-year-old woman with refractory cerebral vasospasm and delayed cerebral ischemia due to aneurysmal subarachnoid hemorrhage, who was successfully treated with mechanical spasmolysis. Through a right-side femoral artery 5F sheath (Radifocus Introducer II 10 cm; Terumo) and after diagnostic angiography confirmed the presence of severe vasospasm of the left middle cerebral artery (MCA), the left internal carotid artery was catheterized with an Envoy 5F MPC guide catheter (Codman/Integra LifeSciences). A Prowler Select Plus microcatheter (Cerenovus) was advanced over a Hybrid007J microguidewire (Balt) through the RHV’s main port (Access-Plus, Merit) to the narrowed M1 segment, after which a pRELAX 4/20 (WallabyPhenox) was advanced into the microcatheter and unsheathed. The stent was deployed for approximately 3 min, after which the pRELAX was pulled through the affected vessels inducing partial vasodilation, as recently described elsewhere (19). At the moment of microcatheter withdrawal, a cloudy content was observed inside the RHV, which resulted in the interruption of the procedure. Both the RHV and the microcatheter were removed and saved for posterior analysis. After a new RHV was connected, the procedure continued with the successful deployment of a pEGASUS-HPC 4.5/30 mm stent (WallabyPhenox) through an Excelsior SL-10 microcatheter (Stryker). The procedure lasted 34 min, during which the patient was heparinized and premedicated with dual antiplatelet treatment (180 mg ticagrelor and 500 mg aspirin PO).

The second case consisted of an 85-year-old male with a history of coronary disease and arterial hypertension scheduled for elective endovascular treatment for symptomatic and severe basilar artery stenosis. Through a right-side femoral artery 5F sheath (Radifocus Introducer II 10 cm; Terumo) and after diagnostic angiography confirmed the presence of severe basilar artery stenosis, the left vertebral artery was catheterized with a Navien A+ 058 125 cm (Medtronic) guide catheter. A Prowler Select Plus microcatheter (Cerenovus) was successfully advanced over a pORTAL 0.014″ microguidewire (WallabyPhenox) through the RHV’s main port (Access-Plus, Merit), crossing the stenotic segment after which a Solitaire AB 4/30 mm stent (Medtronic) was successfully deployed achieving blood flow improvement without signs of extravascular contrast leakage or vascular dissection. Similar to the first case, at the moment of microcatheter withdrawal, a cloudy content inside the RHV was identified, prompting the interruption of the procedure and withdrawal of both the microcatheter and valve for subsequent analysis. After resuming, a final angiographic evaluation ruled out any noticeable occlusive complications. The intervention lasted 63 min, and it was performed while the patient was heparinized and under dual antiplatelet treatment (10 mg prasugrel and 100 mg aspirin PO daily).

### 2.2 *In vivo* analysis

Samples collected from both patients, including the microcatheters, RHV, and their respective cloudy content, were subject to photographic documentation, optical microscopy evaluation (Nikon, SMZ1500), and ATR-FTIR spectroscopy at an independent institution (Institut für Qualitätssicherung und angewandte Schadensanalyse GmbH, Dortmund, Germany). The debris material found inside the RHVs was flushed out using deionized water and then dried. Infrared spectra were then recorded using ATR-FTIR spectroscopy (Alpha spectrometer with ATR diamond crystal unit, OPUS software version 7.5, Bruker) to determine its molecular composition. The infrared spectra were recorded in the mid-infrared range (4,000 to 400 cm^−1^ wavelength) at a resolution of 4 cm^−1^, 24 scans, and using air as background measurement. Samples from the outer surface of the three distinctly colored sections of an additional unused Prowler Select Plus microcatheter (control) were also analyzed. According to the manufacturer, this microcatheter has a proximal nylon (a type of aliphatic polyamide) section and middle and distal hydrophilic sections, identifiable by their different colors. The absorption band spectra of the particulate material were compared with a database and with the spectra found on samples from different portions of the control microcatheter’s outer surface.

### 2.3 *In vitro* analysis

Since debris was found during microcatheters’ withdrawal through the RHVs’ main port, a second independent institution (Euro-Labor GmbH “Institute for Failure Analysis and Materials Investigation,” Bochum, Germany) designed and conducted an *in vitro* experiment to evaluate if this maneuver produced coating delamination. Three unused and sealed 0.021″ inner-diameter microcatheters, two Prowler Select Plus (Cerenovus), and a Trevo Pro 18 (Stryker) were tested. The latter was chosen for comparison due to its similar characteristics (outer hydrophilic coating, similar proximal outer diameter) and because it is one of the most commonly used microcatheters in our institution. The simulation included a 6F Envoy 070 guide catheter (Codman), an Access-Plus RHV (Merit), and a 0.9% isotonic sodium chloride rinsing solution connected through the RHV’s side port. All samples were prepared and manipulated according to their respective manufacturer instructions. After the microcatheters were placed inside the guiding catheter following a standard neurointerventional setup, the RHV’s sealing ring was loosened and tightened two times, and the microcatheters were manually withdrawn once by an interventional neuroradiologist without using a microguidewire. During the simulation, the valves were closed to prevent the backflow of saline solution, but the microcatheters remained movable with an usual amount of force at a relatively standard velocity for all samples. The resulting debris material inside the RHV was flushed out with deionized water, dried out, and then characterized using ATR-FTIR analysis as stated above (Alpha spectrometer with ATR diamond crystal unit, OPUS software version 7.5, Bruker). The source of the debris was assessed by comparing its absorption band spectra with known references and with those found in samples obtained from different sections of a control microcatheter’s outer surface (distal, middle, and proximal portions), similar to the *in vivo* analysis. Samples from the body, sealing ring, and sealing ring’s cap of an unused RHV were also collected and compared.

## 3 Results

### 3.1 *In vivo* analysis

**Figure 1:**
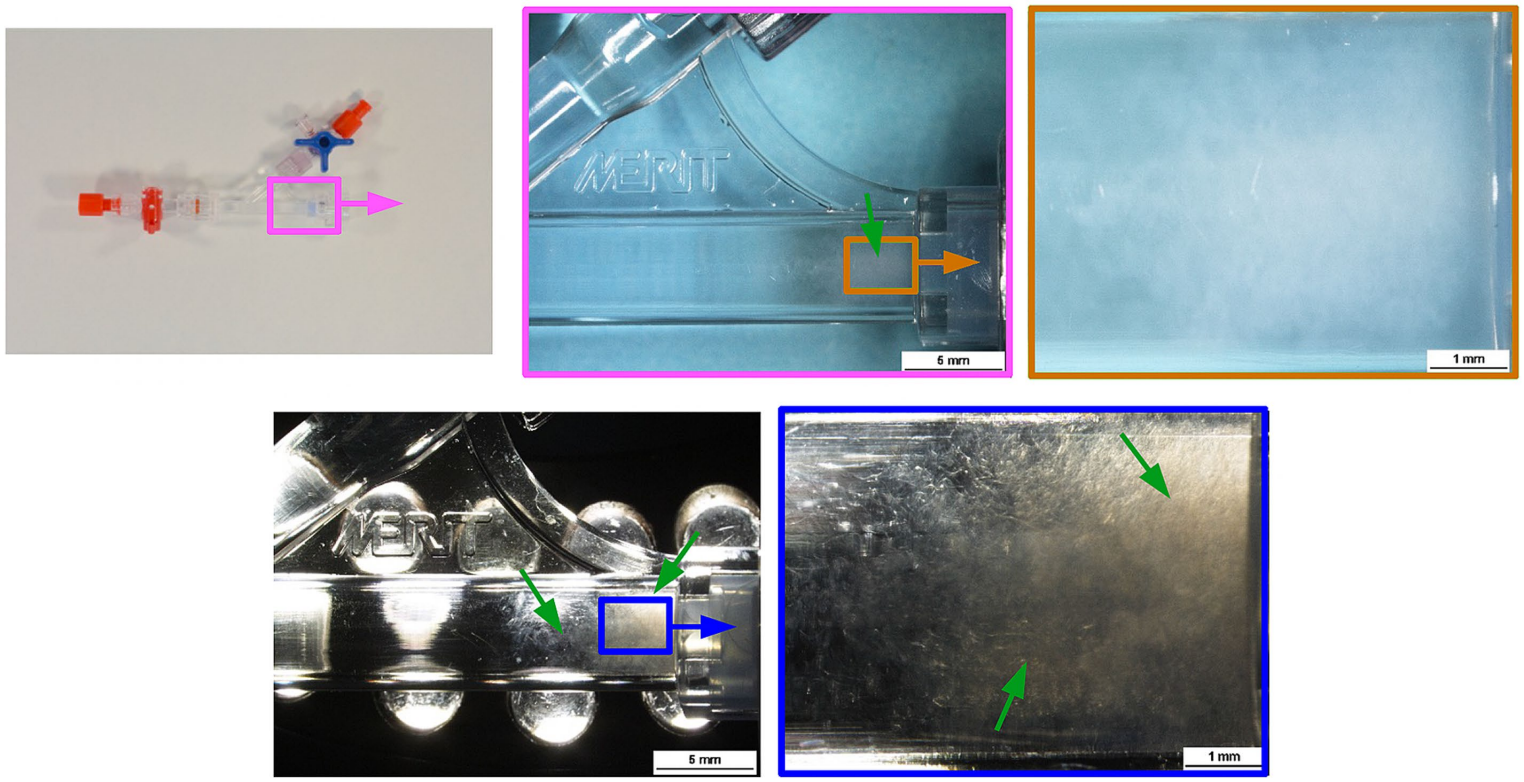
Optical microscopic documentation of *in vivo* observations. During two neurointerventional procedures, the presence of an unusual cloudy-appearing content (green arrows) inside a Merit RHV was identified after a Prowler Select Plus microcatheter was withdrawn. In both cases, the RHV and microcatheters were saved for further analysis in order to identify the nature and origin of the debris. RHV, rotating hemostatic valve.

**Figure 2:**
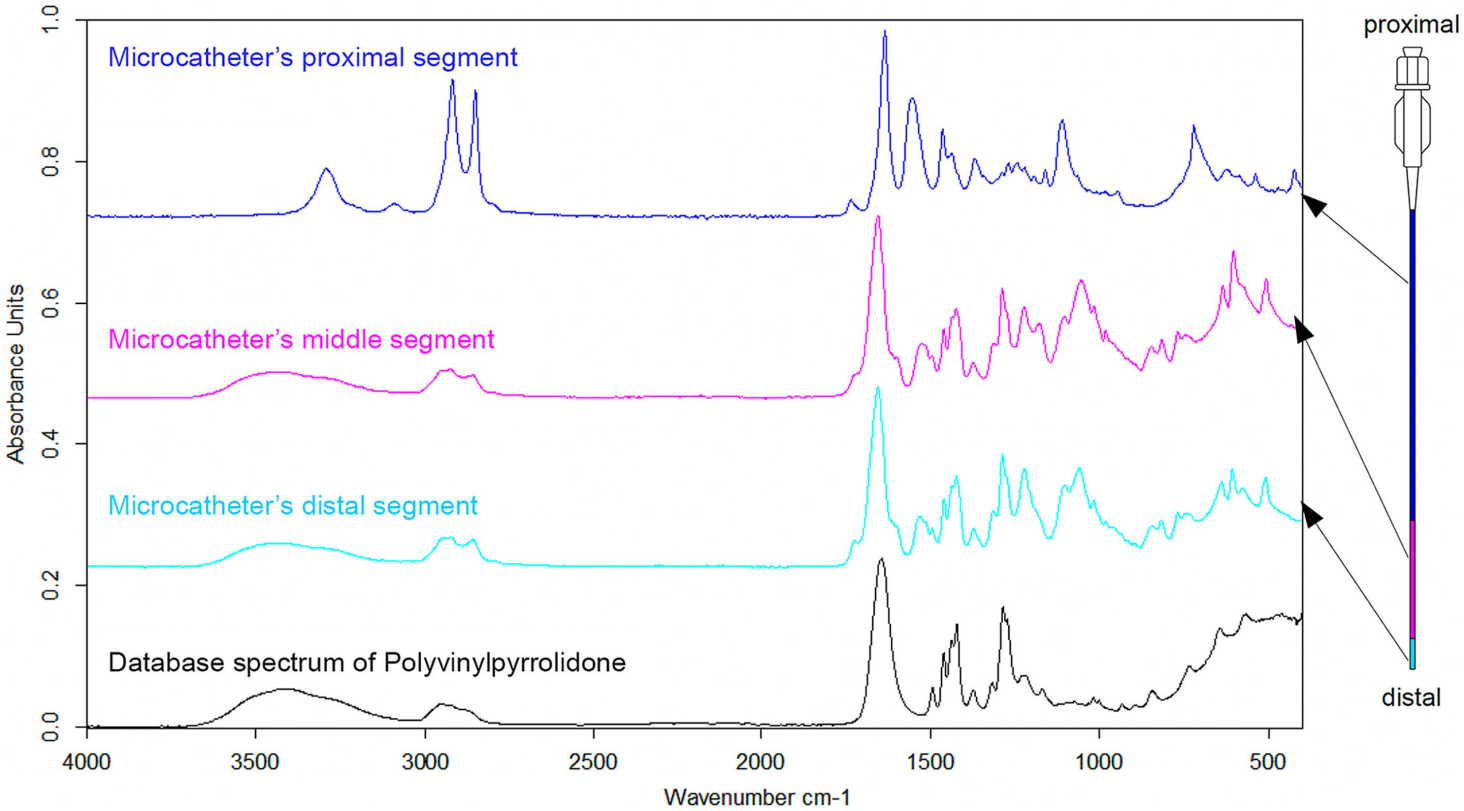
Absorption band spectra from ATR-FTIR spectroscopy - *in vivo* observations. An unused Prowler Select Plus microcatheter was analyzed to identify the components of its outer surface coating. According to the manufacturer’s instructions, the device has three different portions easily identified according to their respective colors. The absorption band spectrum of the outer surface of the distal and middle portions (light blue and magenta segments in this illustration) was identified as PVP, while the spectrum found for the outer surface of the proximal portion (dark blue segment) corresponded to polyamide. ATR-FTIR, attenuated total reflection Fourier-transform infrared spectroscopy; PVP, polyvinylpyrrolidone.

Photographic and optical microscopic documentation revealed cloudy debris inside the RHV after the microcatheter was withdrawn in both patients (Figure 1). In one sample, a slightly larger, 1 mm in diameter isolated particle was found in addition to the cloudy content (Supplementary Figure 1). The ATR-FTIR absorption band spectrum of the outer surface of the distal and middle sections of the unused microcatheter significantly correlated with the database absorption band spectrum of Polyvinylpyrrolidone (PVP), with characteristic vibrational peaks for a wavelength of approx. 1,290 to 1,450 cm^−1^. The absorption band spectrum of the outer surface of the microcatheter’s proximal section was consistent with Polyamide (Figure 2), determined by peaks observed at approx. 1600 to 1,690 cm^−1^, and at approx. 3300 to 3,100 cm^−1^.

**Figure 3:**
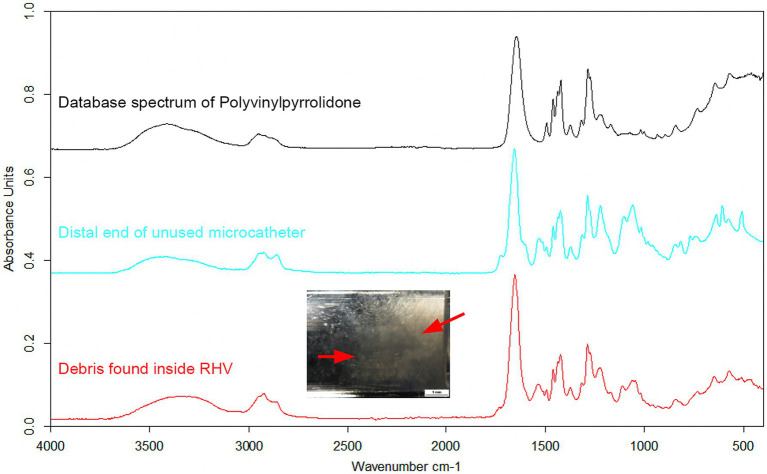
Absorption band spectra from ATR-FTIR spectroscopy – *in vivo* observations. The dried residue found inside the used RHV after the microcatheters were withdrawn (red arrows) significantly correlated with the absorption band spectrum of the outer surface of the distal portion of an un-used Prowler Select Plus microcatheter, which was further identified as PVP according to the database spectrum. ATR-FTIR, attenuated total reflection Fourier-transform infrared spectroscopy; PVP, polyvinylpyrrolidone; RHV, rotating hemostatic valve.

The absorption bands of the dried content observed inside the used RHV were also identified as PVP, similar to the samples obtained from the outer surface of the distal and middle sections of the unused Prowler Select Plus microcatheter (Figure 3). In addition, the ATR-FTIR spectrum of the isolated 1 mm particle matched the spectra of human proteins in the database (Supplementary Figures 1, 2).

### 3.2 *In vitro* analysis

**Figure 4:**
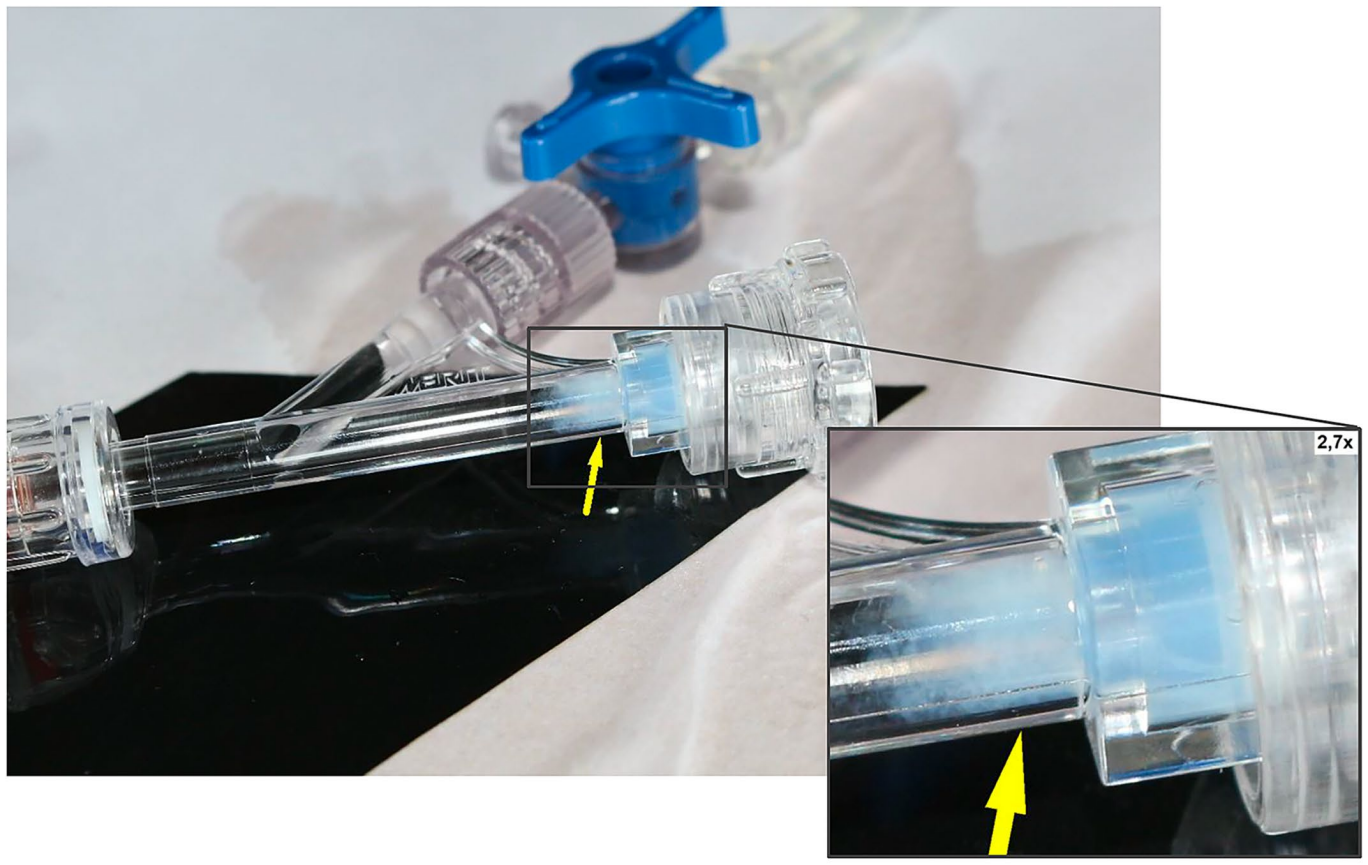
*In vitro* experiment. A standard neurointerventional setup was simulated using a 6F Envoy 070 guiding catheter (Codman), an Access-Plus RHV (Merit), and one of two different 0.021″ inner-diameter microcatheters: Prowler Select Plus (Cerenovus) and Trevo Pro 18 (Stryker). A flushing system using 0.9% isotonic sodium chloride solution was connected to the side port of the RHV. After the RHV’s sealing ring was loosened and tightened two times, both microcatheter types were withdrawn once through the RHV’s main port without using a microguidewire. A cloudy-appearing content was identified inside the RHV after withdrawing the Prowler Select Plus microcatheter (yellow arrow), which was later identified as PVP through ATR-FTIR spectroscopy. ATR-FTIR, attenuated total reflection Fourier-transform infrared spectroscopy; PVP, polyvinylpyrrolidone; RHV, rotating hemostatic valve.

**Figure 5:**
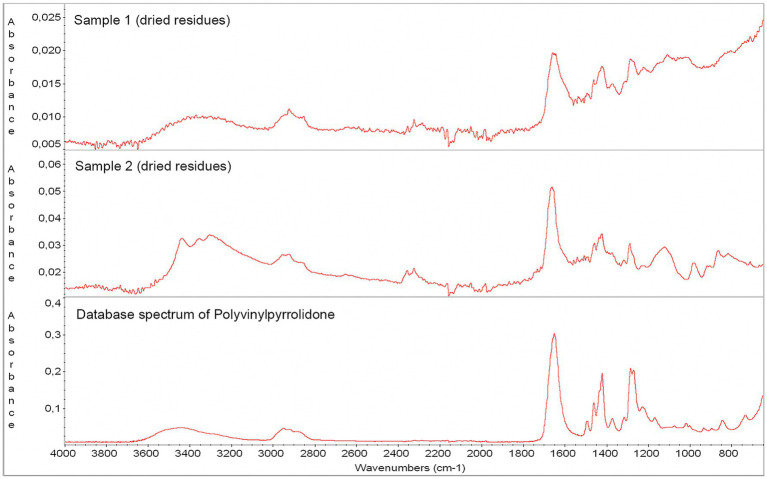
Absorption band spectra from ATR-FTIR spectroscopy – *in vitro* observations. The dried residues found inside the RHV after withdrawing two previously unused Prowler Select Plus microcatheters (Samples 1 and 2) were analyzed. In both cases, the absorption band spectrum corresponded to PVP. ATR-FTIR, attenuated total reflection Fourier-transform infrared spectroscopy; PVP, polyvinylpyrrolidone; RHV, rotating hemostatic valve.

The *in vitro* simulation of the microcatheter withdrawal maneuver produced results similar to those of the *in vivo* observations, particularly when testing the Prowler Select Plus microcatheters. In both samples, a cloudy content was observed inside the RHV near its sealing ring after these microcatheters were removed through the RHV’s main port (Figure 4; Supplementary Video 1). On the contrary, no residues were noted after withdrawing the Trevo Pro 18 microcatheter (Supplementary Figure 3; Supplementary Video 2). The ATR-FTIR spectra of the dried debris from the first two samples closely matched the database spectrum of PVP (Figure 5).

**Figure 6:**
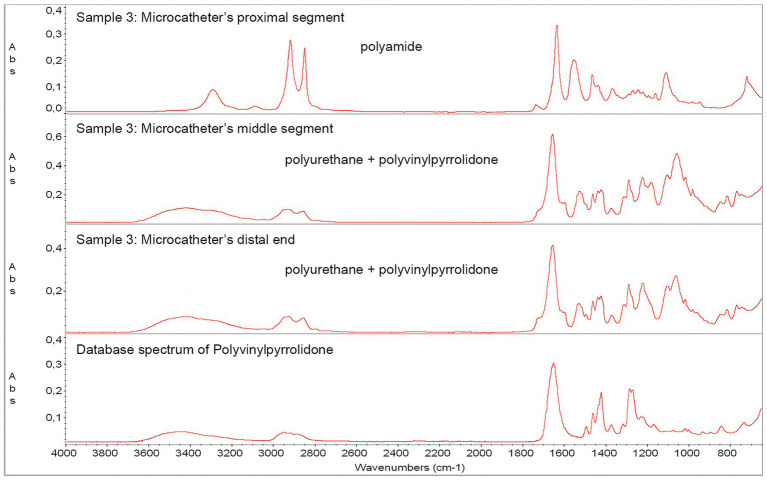
Absorption band spectra from ATR-FTIR spectroscopy - *in vitro* observations. An unused Prowler Select Plus microcatheter was analyzed to identify the components of its outer surface coating. The absorption band spectrum of the outer surface of the distal and middle portions was identified as a compound material consisting of polyurethane and PVP, while the spectrum found for the outer surface of the proximal portion corresponded to polyamide. ATR-FTIR, attenuated total reflection Fourier-transform infrared spectroscopy; PVP, polyvinylpyrrolidone.

The ATR-FTIR spectrum of the outer surface samples taken from the middle and distal portions of the Prowler Select Plus was identified as a compound material consisting of polyurethane and PVP. In contrast, the spectrum of the outer surface of the microcatheter’s proximal portion corresponded to polyamide (Figure 6). The ATR-FTIR spectra of the body, sealing ring, and sealing ring’s cap of the RHV (Supplementary Figures 4, 5) corresponded to the database spectra of polycarbonate, silicone, and polyoxymethylene, respectively.

## 4 Discussion

The presence of a cloudy-appearing liquid content inside the RHV after performing a commonly used microcatheter withdrawal maneuver forced the two endovascular procedures to be interrupted. In both cases, a Prowler Select Plus microcatheter and a Merit RHV were being used. ATR-FTIR spectra of the dried residues found inside the RHV, as well as the spectrum of the outer surface of the distal and middle portions of an unused control Prowler Select Plus microcatheter, showed a significant accordance with PVP. An *in vitro* simulation replicated this phenomenon after using the same microcatheters; however, this was not the case for the Trevo Pro 18. A second and independent ATR-FTIR spectroscopy analysis determined that these residues also corresponded to PVP and appeared to originate from the outer layer of the middle and distal portions of the Prowler Select Plus, and did not seem to be related to the RHV.

Most devices currently in use for intravascular neurointerventional procedures employ lubrication coatings made from hydrophilic and/or hydrophobic polymers, such as PVP, polyacrylic acid, polytetrafluoroethylene, and silicone. Polymer coatings can effectively reduce friction between the device’s surface against other devices or against blood vessel walls, favoring intravascular maneuverability, which in turn reduces the risk of endothelial damage, thrombosis, and vasospasm (4, 20). However, recent evidence suggests that fragments of polymer coating can detach and enter the patient’s circulation, leading to severe outcomes such as pulmonary embolism, ischemic stroke, myocardial infarction, tissue necrosis, and death (6, 7, 9, 21–23). In 2015, the US Food and Drug Administration (FDA) issued a safety warning about lubricious coating embolism due to the increasing number of reported cases. However, this report did not identify any particular device manufacturer or brand responsible for having a higher risk of HCE (7). More recently in neurointervention, along with ischemic and hemorrhagic complications, HCE has been presumed to be among the potential causes of delayed-onset non-ischemic cerebral enhancing (NICE) lesions (24). This rare radiological finding can be asymptomatic or present with severe morbidity, often requiring long-term pharmacological treatment (25).

There are several reasons why coating delamination can occur, including factors related to device design, manufacturing, and clinical use. Mechanical abrasion occurs when the device comes into contact with vessel walls or other devices, generating an incremental strain on chemical bonds within the polymer structure and between the polymer and the device surface. Upon reaching a bond-energy threshold, the chemical bonds break, resulting in coating separation. Excess friction can result from navigating a device through tortuous, atherosclerotic, narrow vessels or acutely angulated bifurcations (18). Time-dependent chemical degradation of devices exposed to saline or blood has also been reported as an additional cause for coating failure (1, 26), and manufacture-related factors, such as specific device types, coating compositions, proprietary additives, curing processes, coverage styles, and techniques to modify surface substrates, also impact coating integrity (15, 21, 27). Perhaps more important for neurointerventionalists, certain commonly used endovascular techniques, such as manipulating guiding sheaths and microcatheters (3, 6, 15, 17), tight-fitting coaxial and triaxial systems (13, 14), and inserting or torquing microguidewires (10, 13, 16), have also been associated with coating dislodgement. Furthermore, improper handling or use of devices, including incorrect sizing or reshaping and the use of damaged or expired devices, can also contribute to this issue (1).

To our knowledge, no clinical reports have yet demonstrated a similar phenomenon under these circumstances, and only one *in vitro* study has examined the potential relationship between HCE and microcatheter manipulation through RHVs. Kan et al. tested the Excelsior SL-10 (Stryker), Headway 17 (MicroVention), and Echelon 10 (Medtronic) microcatheters in different setups and combinations with different port insertion configurations and RHV types (Abbott and Boston Scientific), and revealed that microcatheter insertion through the side port of Abbott RHVs induced coating damage and debris dislodgement. Similar to our results, the authors detected PVP by conducting ATR-FTIR spectroscopy debris analysis (17). PVP is an FDA-approved material commonly used as part of the outer coating of many interventional devices, and although generally considered safe, its presence in cases affected by HCE has been reported (6, 14). The precise cause behind the coating delamination observed in the two cases presented in this study has not yet been determined. Neurointerventional procedures at our institution adhere to the device instructions from each manufacturer and FDA recommendations to reduce the risk of HCE (7), and unlike in Kan et al.’s study, the microcatheters were manipulated through the RHV’s main port without any other devices being inserted into their side port, preventing unnecessary friction. Both procedures were uneventful until the cloudy material was recognized, and they were completed within the average time frames, making an abnormal chemical interaction with saline or blood products less likely.

Depending on the specific information required, a range of analytical techniques can be employed to identify and characterize material attributes (28). ATR-FTIR spectroscopy is a highly useful analytical technique for identifying unknown organic and polymeric materials, detecting a unique set of absorption bands that act like a compound’s unique fingerprint. Analyzing these bands can confirm the identity of constituent elements and chemical structure in the surface regions of biomedical coatings (29, 30) or detect the presence of specific impurities, such as human proteins detected in one of the samples obtained from one patient (Supplementary Figures 1, 2). Our findings indicated a definite match between the debris composition and the material obtained from the outer surface of the distal and middle portions of the Prowler Select Plus microcatheter. Although PVP is a common hydrophilic polymer used by different neurointerventional manufacturers, the only devices that were utilized in both patients were the microcatheters, RHVs, and femoral sheaths. In this study, we did not test the femoral sheaths; however, they never came into direct contact with the RHV during the procedures, which was the location where this unusual cloudy content was initially observed. Additionally, the samples collected from different parts of the RHV did not match the ATR-FTIR absorption band spectrum of the debris in question. In theory, the identified debris could have also originated from the inner surface of the guiding catheters used, Envoy and Navien. However, according to their respective manufacturers, both have a lubricious PTFE-lined inner lumen. While we did not conduct a spectroscopic analysis of these devices, the combination of our experimental data and the existing theoretical information suggests that the outer surface of the distal and middle sections of the Prowler Select Plus microcatheter is likely the source of the abnormal material found within the RHVs.

It is also important to note that it remains uncertain whether this phenomenon resulted in clinical consequences. In the first case, ischemic complications were attributed to the known unfavorable progression of refractory vasospasm, while for the second, there has been less than a year of radiological and clinical follow-up at the time of writing this manuscript. Neither have yet been diagnosed with NICE lesions.

### 4.1 Future directions and recommendations

While our report is based on a small number of patients and samples, the discovery of PVP delamination during the withdrawal of the Prowler Select Duo microcatheter is a matter of concern. Any potential coating defect in a widely used intravascular catheter could put an unknown number of patients at risk of HCE. Therefore, as other authors have already suggested regarding this topic (8, 25), further studies comparing currently available devices with transparent, publicly available results are necessary. Intravascular interventional instruments such as microcatheters are considered Class III medical devices in the European Union and China and Class II devices in the US, meaning they do not require clinical trials before being approved for clinical use (26). Furthermore, the FDA recommends that manufacturers themselves are responsible for developing appropriate particulate matter assessment procedures and providing an interpretation of their results (7, 31); however, despite the existence of standard methods for particulate detection, no size or number thresholds have been proposed (26). Ultimately, the overall benefit of lubricious-coated devices appears to outweigh the associated risks; however, until better data surfaces, neurointerventionalists should ensure proper device use, storage, and careful manipulation during endovascular procedures (7). Based on our current findings, we believe monitoring the RHV during microcatheter manipulation is essential, particularly during the withdrawal phase. Furthermore, aspiration during this maneuver may be critical in preventing the embolization of coating particulates that may not be easily visible to the naked eye.

### 4.2 Limitations

This preliminary study began after identifying potential coating delamination during the treatment of two patients. As a result, an *in vivo* control subject was not included. Since the main objective of this report was to determine the molecular composition and source of the debris, no further analysis was conducted outside ATR-FTIR spectroscopy. Even though the *in vitro* experiments were methodically carried out in the same way, three major limitations could limit their significance: (1) the speed of withdrawal was not measured; (2) the slightly different outer diameter of the microcatheters may influence the friction; and (3), the tightness of RHV, which alters the friction during inserting and withdrawing of the microcatheter, was not measured. In summary, the force required to withdraw the catheter could have been measured and adjusted. For pragmatic reasons, we have focused on the typical clinical application of microcatheters; however, we acknowledge that the small sample size and the use of non-standardized measurements limit the validity and generalizability of our findings. Consequently, it remains unclear whether this phenomenon was caused by a manufacturing defect in the device or by factors related to manipulation. Despite these limitations, our study offers preliminary insights that call for further investigation.

## 5 Conclusion

Although polymer coatings improve intravascular device maneuverability and reduce the risk of vessel injury, they can also detach and embolize into the intracranial circulation, posing a risk of severe complications. The presence of a cloudy liquid content inside the RHV during microcatheter manipulation may serve as an indication of coating delamination. In particular, we confirmed the presence of PVP debris inside the RHV after withdrawing the Prowler Select Plus microcatheter and traced their origin back to the surface coating of the distal and middle portions of the microcatheter. Despite the associated costs and publishing barriers, future research should strive to compare a broader range of devices under stricter analytical conditions, including conducting automated lubricity-durability and particulate generation analyses.

## Data Availability

The datasets presented in this article are not readily available due to patient privacy protection. Requests to access the datasets should be directed to the first author SM, sebastian.j.mueller@gmail.com.
