# Supplementary material for: Hydrophilic polymer coating delamination during neurointerventional treatment after microcatheter withdrawal: particulate identification through attenuated total reflection Fourier-transform infrared spectroscopy
Source: Front Neurol. 2025 Jan 15;15:1479375. doi: 10.3389/fneur.2024.1479375 (PMC11774725; doi:10.3389/fneur.2024.1479375)
Supplement: Supplementary file 3 [file Data_Sheet_1.pdf]

Supplementary Material

1 Supplementary Figures

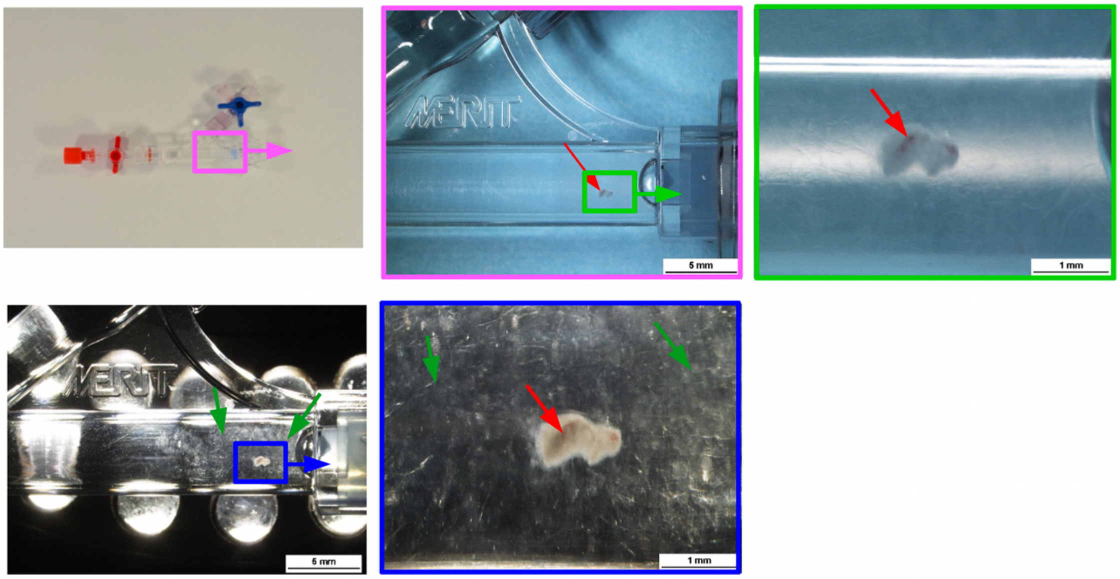

Figure 1. Optical microscopic documentation of *in vivo* observations – isolated particle. In one of the neurointerventional procedures, the presence of a slightly larger, 1mm whitish isolated particle (red arrows) besides the already discussed unusual cloudy-appearing content (green arrows) was identified inside a Merit RHV after a Prowler Select Plus microcatheter was withdrawn. RHV, rotating hemostatic valve.

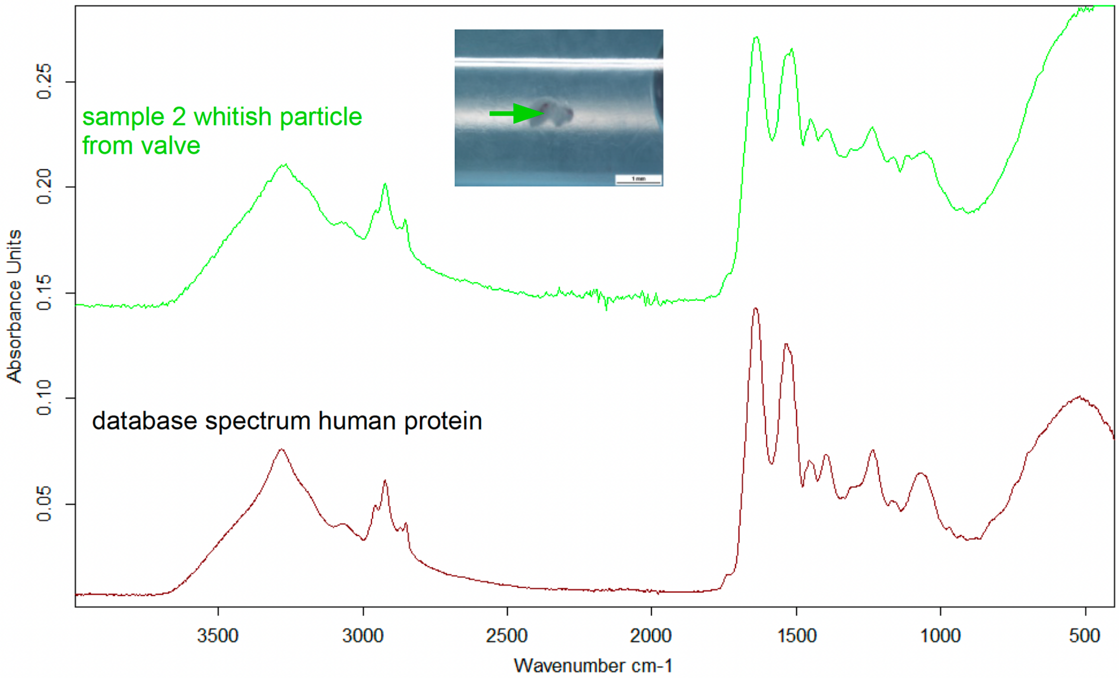

Figure 2. Absorption band spectra from ATR-FTIR spectroscopy – *in vivo* observations. The whitish isolated particle found inside one of the two used RHV after the microcatheter was withdrawn (red arrows) correlated with the absorption band spectrum of different human proteins. Importantly, it differed from the absorption band spectrum of the dried cloudy material also found in the same sample, later identified as PVP. ATR-FTIR, attenuated total reflection Fourier-transform infrared spectroscopy; PVP, polyvinylpyrrolidone; RHV, rotating hemostatic valve.

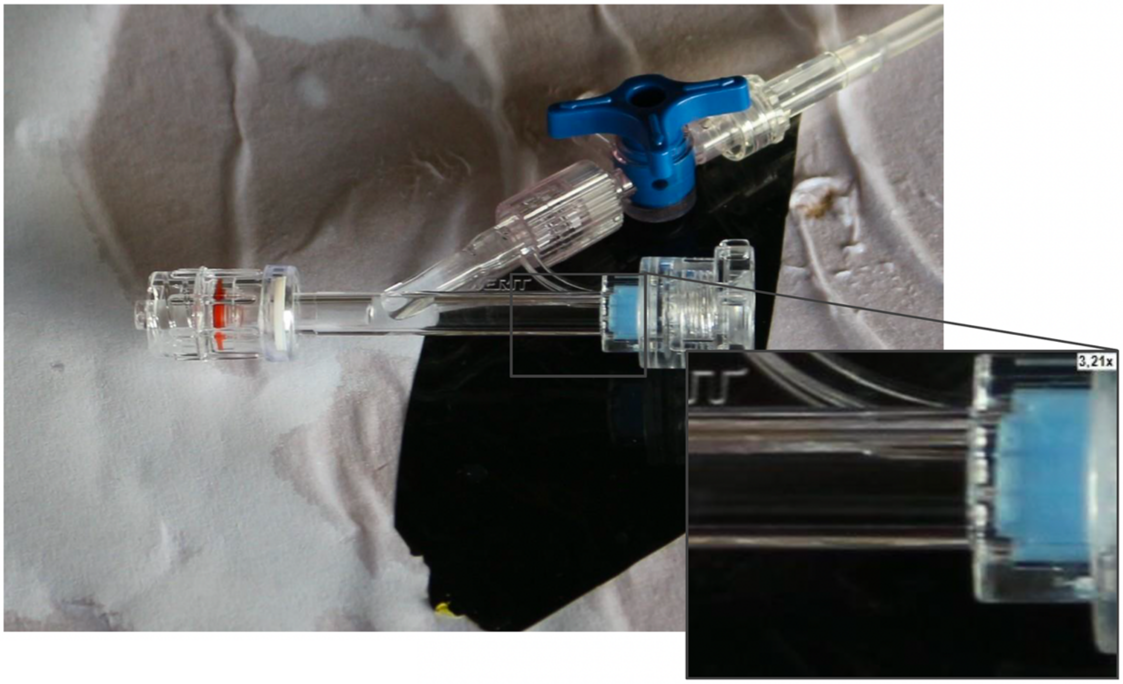

Figure 3. *In vitro* simulation - Trevo Pro 18 microcatheter. After the RHV's sealing ring was loosened and tightened two times and the Trevo Pro 18 (Stryker) was manually withdrawn through the RHV's main port, no signs of debris were observed inside the RHV. The maneuver was conducted without using a microguidewire and with a flushing system using 0.9% isotonic sodium chloride solution connected to the side port of the RHV. RHV, rotating hemostatic valve.

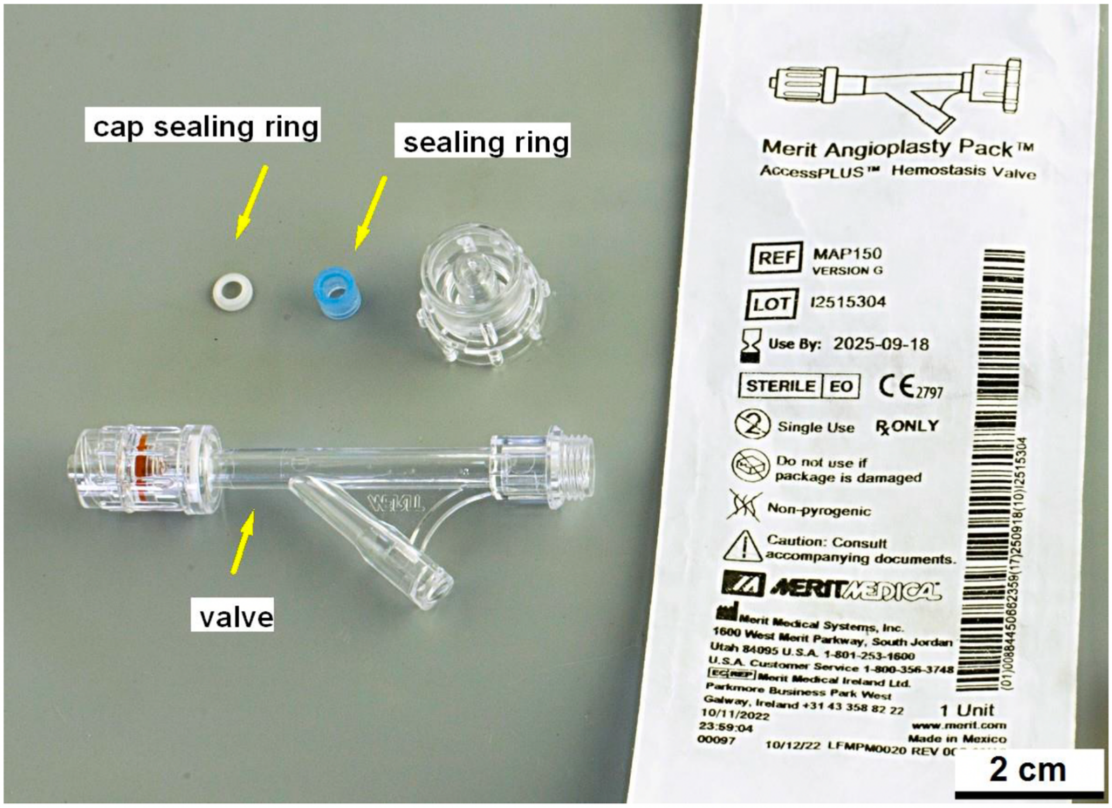

Figure 4. *In vitro* simulation - RHV parts. A new, unused Access-Plus RHV (Merit) was used for the in vitro simulation, and subsequently, each part was analyzed with ATR-FTIR spectroscopy. ATR-FTIR, attenuated total reflection Fourier-transform infrared spectroscopy; RHV, rotating hemostatic valve.

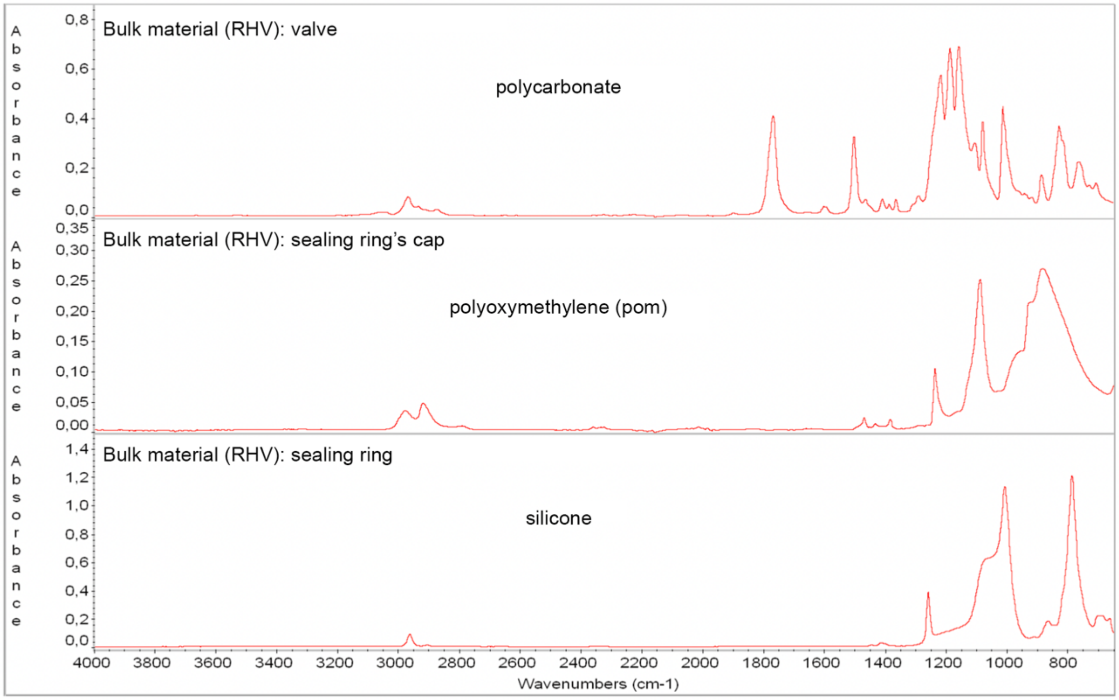

Figure 5. Absorption band spectra from ATR-FTIR spectroscopy - *in vitro* observations. The ATR-FTIR spectra of the body (valve), sealing ring, and sealing ring's cap of the RHV corresponded to the database spectra of polycarbonate, silicone, and polyoxymethylene, respectively. ATR-FTIR, attenuated total reflection Fourier-transform infrared spectroscopy; RHV, rotating hemostatic valve.

2 Supplementary videos

Video 1. *In vitro* simulation – Prowler Select Plus microcatheter. After manually withdrawing the microcatheter, a particulate content was clearly seen inside the RHV.

Video 2. *In vitro* simulation - Trevo Pro 18 microcatheter. After manually withdrawing the microcatheter, no signs of debris were observed inside the RHV.
